# Liquid biopsy evaluation of circulating tumor DNA, miRNAs, and cytokines in meningioma patients

**DOI:** 10.3389/fneur.2023.1321895

**Published:** 2024-01-08

**Authors:** Veronica Aran, Renan Lyra Miranda, Manoela Heringer, Anna Carolina Carvalho da Fonseca, Felipe Andreiuolo, Leila Chimelli, Sylvie Devalle, Paulo Niemeyer Filho, Vivaldo Moura-Neto

**Affiliations:** ^1^Laboratório de Biomedicina do Cérebro, Instituto Estadual do Cérebro Paulo Niemeyer, Rio de Janeiro, Brazil; ^2^Neuropathology and Molecular Genetics Laboratory, Instituto Estadual do Cérebro Paulo Niemeyer, Rio de Janeiro, Brazil; ^3^Rede D'Or, IDOR - Instituto D'Or de Pesquisa e Ensino, Rio de Janeiro, Brazil; ^4^Neurosurgery Division, Instituto Estadual do Cérebro Paulo Niemeyer, Rio de Janeiro, Brazil

**Keywords:** liquid biopsy, meningioma, ctDNA, miR-21, cytokines, IL-6 liquid biopsy, IL-6

## Abstract

**Introduction:**

Liquid biopsy is a non-invasive method used to detect cancer and monitor treatment responses by analyzing blood or other bodily fluids for cancer biomarkers. Meningiomas are the most common primary central nervous system tumors, and biomarkers play a crucial role in their diagnosis, prognosis, and treatment monitoring. The World Health Organization (WHO) classifies meningiomas based on tumor grades and molecular alterations in genes such as in NF2, AKT1, TRAF7, SMO, PIK3CA, KLF4, SMARCE1, BAP1, H3K27me3, TERT promoter, and CDKN2A/B. Liquid biopsy, specifically cell-free DNA (cfDNA) analysis, has shown potential for monitoring meningiomas as it can detect ctDNA release in the blood, unaffected by the blood-brain barrier. MicroRNAs (miRNAs) have also been found to be deregulated in various cancers, including meningiomas, presenting potential as diagnostic biomarkers. Additionally, studying cytokines in the tumor microenvironment may aid in establishing prognostic or diagnostic panels for meningiomas.

**Methods:**

In the present study we analyzed the DNA coming from both the plasma and tumor samples, in addition to analyze miRNA-21 and cytokines in the plasma of 28 meningioma patients.

**Discussion and Conclusion:**

Our findings indicate that the detection of ctDNA in the plasma of meningioma patients is feasible. However, it's important to note that certain challenges persist when comparing plasma DNA analysis to that of tumor tissues. In our study, we observed a paired identification of mutations in only one patient, highlighting the complexities involved. Furthermore, we successfully identified miR-21 and cytokines in the plasma samples. Notably, our analysis of Interleukin 6 (IL-6) unveiled higher expression in the clear cell subtype compared to the other types. Despite the ongoing research, the clinical implementation of liquid biopsy in meningiomas remains somewhat limited. Nevertheless, our promising results underscore the need for further investigation.

## Introduction

The methodology known as liquid biopsy represents a non-invasive method for detecting cancer and monitoring treatment responses by analyzing blood or other fluids, such as urine, cerebral spinal fluid and saliva, in order to search for relevant cancer biomarkers (1). Examples include cell-free DNA (cfDNA), circulating tumor DNA (ctDNA), circulating tumor cells (CTCs), microRNAs (miRNAs), and inflammatory cytokines. Liquid biopsy can be useful to investigate distinct tumors such as the ones affecting the central nervous system (CNS).

The most common primary CNS tumors are meningiomas, which are frequently benign, arising from meningothelial (arachnoid) cells (2). The WHO categorization of malignancies of the CNS, released in 2021, consider meningiomas a single tumor type, with three grades and broad morphological spectrum including 15 subtypes (3). For example, Grade I meningiomas (Benign) include the subtypes: Meningothelial, Fibrous Meningioma, Transitional Meningioma and Psammomatous; Grade II Meningiomas include the subtypes: Atypical Meningioma; Grade III Meningiomas (Malignant) include Anaplastic Meningioma. The subtype and grade of a meningioma plays a pivotal role in shaping treatment approaches and forecasting patient prognosis (2). The WHO classification incorporates certain molecular alterations associated with meningioma clinicopathologic utility, and the genes affected include: *NF2, AKT1, TRAF7, SMO, PIK3CA; KLF4, SMARCE1, BAP1; H3K27me3; TERT* promoter, *CDKN2A/B* in CNS WHO grade 3 (3). On the basis of the involvement of the Neurofibromatosis type 2 (NF2) gene, which is involved in tumor suppression, the genomic landscape of meningiomas can be generally divided into two subsets: tumors linked to NF2 mutations and tumors with mutations unrelated to NF2 (4). In addition, there are different molecular alterations linked to the different meningioma Grades. Interestingly, meningiomas present mutations in *NF2* irrespectively of the histological grade. Grade I tumors show alterations in *TRAF7, SMO*, and *PIK3CA* and Grade II was also shown to present alterations in *TRAF7* (2). In the present study we focused our ctDNA analysis in searching for mutations in the genes *NF2*, phosphatidylinositol-4,5-bisphosphate 3-kinase catalytic subunit α (PIK3CA), frizzled class G protein-coupled receptor (SMO) and v-Raf murine sarcoma viral oncogene homolog B1 (BRAF), the latter although rare, has been reported in rhabdoid meningiomas (5, 6).

Meningiomas are typically diagnosed using imaging techniques such as magnetic resonance imaging (MRI) or computed tomography (CT) scans and despite their mostly benign nature, meningiomas can cause significant patient morbidity (2). Current research aims to discover new biomarkers and create non-invasive therapies for meningiomas. Liquid biopsy, though actively researched, has limited clinical use for meningiomas. While circulating tumor cells (CTCs) are uncommon in meningioma patients' blood, circulating free DNA (cfDNA) can be detected, enabling monitoring of disease progression and treatment response. This is facilitated by the unique characteristic of meningiomas, where the blood-brain barrier doesn't hinder the release of circulating tumor DNA (ctDNA) into the bloodstream. For example, in a proof-of-concept study, ctDNA was detected in the blood from 15 patients containing variable mutations (7). Another recently published study reported liquid biopsy as a potential tool for diagnosing and predicting outcomes in meningioma patients (8).

In addition to DNA-level changes, tumors can exhibit variations in microRNA (miRNA) expression, which are often found deregulated in various cancers and can be negatively or positively controlled, functioning as either onco-miRNAs or tumor suppressors (9, 10). MiRNAs, ~22-nucleotide small non-coding RNAs, are crucial in regulating gene expression. They play a role in post-transcriptional regulation of mRNAs, impacting developmental timing, apoptosis, cellular hematopoietic differentiation, proliferation, and organ development (11). Several studies have utilized miRNAs as diagnostic biomarkers alone or in conjunction with other known biomarkers (12). MiRNAs can be detected in bodily fluids, such as blood, making them valuable cancer biomarkers with significant potential in diagnosis and prognosis (13). MiR-21 was the first reported miRNA involved in cancer, with overexpression reported in multiple cancers and associated with high oncogenic properties, influencing various cellular processes from tumor initiation to cell death. Additionally, it has been implicated in cancer treatment resistance. In meningiomas, miR-21 has been reported in cancerous tissues and more advanced tumor cases (14, 15).

Cytokines are also under investigation as biomarkers for several cancers, being detected as a panel of different cytokines or combined with other relevant molecules (16). The composition and influence of meningioma's tumor microenvironment are not fully understood. Infiltration of immune cells and their polarization toward pro-tumor phenotypes are observed in higher-grade tumors (II and III) (17, 18). In this context, studying cytokines can be of great value, particularly those that attract the cells of the immune system and those related to the immunosuppression (19, 20). Thus, we consider that the measurement of these cytokines in circulating blood can contribute to establishing an improved monitoring of meningiomas.

In this current study, we examined cfDNA extracted from both plasma and tumor samples in an attempt to match the same mutations in both samples. Additionally, we investigated the presence of miR-21 and the different cytokines: Interleukin-6 and Interleukin-10 (IL-6 and IL-10), Granulocyte-Macrophage Colony-Stimulating Factor (GM-CSF) and Chemokine (C-C motif) Ligand 2 (CCL2) in the plasma of 28 patients diagnosed with meningioma.

## Materials and methods

### Study population

Twenty-eight adult patients with meningioma (WHO grades I and II only, since there was no patient presenting grade III meningioma during the period analyzed) were included in the analysis after histopathological confirmation during the year of 2022. Written informed consent was obtained from each patient by the Human Ethics Committee of the *Instituto Estadual do Cérebro Paulo Niemeyer* (protocol No. CAAE 90680218.6.0000.8110).

### DNA extraction from formalin-fixed paraffin-embedded blocks

The formalin-fixed paraffin-embedded (FFPE) blocks containing the tumor samples were used to create slides, each consisting of two sections measuring 5 μm in thickness. In total, 4–10 sections were used for DNA extraction, depending on the size of the section, using the ReliaPrep FFPE gDNA Miniprep System kit (Promega Corporation, Madison, USA), following the manufacturer's guidelines. DNA concentration was then quantified using the Quantus™ Fluorometer (Promega Corporation, USA).

### cfDNA extraction and quantification

Liquid biopsy was conducted to collect patient samples. Peripheral blood from the patients was obtained through venipuncture immediately prior to surgery, yielding a total of 8 mL of blood using EDTA tubes. Two successive centrifugation steps were performed to separate plasma from serum. The first centrifugation was carried out at 1,200 × g for 10 min, followed by a second centrifugation at 16,000 × g for 15 min at 4°C. Subsequently, 1 mL of the plasma was processed using the Maxwell^®^ RSC automated instrument from Promega Corporation (USA,) following the manufacturer's protocols in order to isolate cfDNA from the plasma. The cfDNA samples were then quantified using the Quantus™ Fluorometer (Promega Corporation, USA).

### Droplet digital PCR analysis

The droplet digital PCR (ddPCR) was employed to investigate the presence of ctDNA in the patient's plasma using Bio-Rad's mutation-specific assays (previously validated by Bio-Rad) targeting the wild-type and mutant versions of the following genes: NF2 R57^*^, BRAF V600 Screening Kit (*V600E, V600K, and V600R*, plus wild-type), *PIK3CA E545K, SMO L412F, and SMO W535L*, which have been described in meningioma tumors. PCR reactions were conducted using a 20 μL reaction mixture consisting of 10 μL of ddPCR supermix (without dUTP; Bio-Rad, CA, USA), 1 μL of 900 nmol/L primers/250 nmol/M probes, and up to 9 μL of DNA and RNA-se free water. Droplets were generated and analyzed using the QX200 Droplet Digital PCR System (Bio-Rad, USA). The amplification process followed specific cycling conditions: an initial cycle at 95°C (2°C/s ramp) for 10 min, followed by 40 cycles at 94°C (2°C/s ramp) for 30 s, 55°C for 1 min, and a final cycle at 98°C (2°C/s ramp) for 10 min. The reaction mixture was then held at 4°C until processing. Absolute quantification of mutant and wild-type alleles was determined through Poisson distribution analysis using QuantaSoft Analysis Software version 1.6.6 (Bio-Rad). Thresholds for allele detection were established based on the signal obtained from empty droplets (negative control), as specified by the manufacturer (Bio-Rad). The percentage of mutant alleles was calculated using the QuantaSoft Analysis Software. To maintain quality control, negative controls were included in all the runs to identify and exclude potential contamination artifacts while ensuring proper gating. In addition, extra negative control tests were always performed for each assay that we used. Furthermore, a validation test was performed with a known mutant tumor sample to identify the limit of detection of low frequency mutations using ddPCR, and the results suggested that a variant allele frequency (VAF) over 0.04% should be considered positive for mutation (data not shown).

### MicroRNA extraction and quantification

Extraction and isolation of microRNA were performed using the Maxwell^®^ RSC miRNA Plasma and Serum Kit (Promega) on the Maxwell^®^ RSC Instrument (Promega), following the manufacturer's instructions, using 300 μL of plasma in each extraction. The sample quantification was estimated using the Quantus™ Fluorometer, pre-programmed with RNA quantification protocols using the QuantiFluor^®^ RNA System kit (Promega Corporation, USA).

To convert the extracted RNA into cDNA, the TaqMan^®^ Advanced miRNA cDNA Synthesis Kit (Thermo Fisher Scientific) was used, following the manufacturer's instructions. In addition, ddPCR reaction was performed for miR-21 evaluation, where a CNV reaction was performed using pre-designed master mix and probes (Bio-Rad, USA) on the QX200 Droplet Digital PCR System.

### Enzyme-linked immunosorbent assay

Cytokines IL-6, IL-10, GM-CSF, and CCL2 levels in the plasma were analyzed using 50 μL of sample and human ELISA kits from R&D systems, according to the manufacturer's instructions. The absorbance at 450 nm was evaluated using an EPOCH microplate spectrophotometer (BioTek Instruments, Winooski, VT, USA) and the ELISA plate reader was Victor X3 (PerkinElmer, USA). Values below the limit of detection were considered as zero for analysis purposes.

### Statistical analyses

Regarding the statistical analyses, for ELISA assays, the Mann-Whitney tests were performed using GraphPad Prism 9 and *p*-values were obtained from the same software and considered significant when *p* < 0.05.

## Results

### Clinicopathological characteristics of the cohort

The study included 28 paired tumor and plasma samples from patients with meningioma WHO grades I (19/28) and II (9/28). Within WHO grade I group the meningioma subtypes Transitional and Meningothelial represented 52.63 and 31.58% of the total, respectively. The subtypes Metaplastic and Angiomatous were the minority (one case each). In one case, determining the precise meningioma subtype was hindered by the presence of neurosurgical cauterization artifacts. Patients median age was 60 years (36–75), being 60.0 (42–75) for females and 60.5 (36–70) for males. Most of the patients were female (71.43%). Clinicopathological characteristics are summarized in Table 1.

**Table 1:** Overall clinical characteristics of the patients.

**Clinical variables**	***N* (%)**
**Sex**	
Male	8 (28.57%)
Female	20 (71.43%)
**Median age, yrs. (range)**	60 (36–75)
Male	60.5 (36–70)
Female	60.0 (42–75)
**WHO grade I**	19 (100%)
Transitional	10 (52.63%)
Meningothelial	6 (31.58%)
Metaplastic	1 (5.26%)
Angiomatous	1 (5.26%)
Undetermined	1 (5.26%)
**WHO grade II**	9 (100%)
Atypical	8 (88.89%)
Clear cell	1 (11.11%)

### Analyses of DNA from paired plasma and tumor tissue

**Figure 1:**
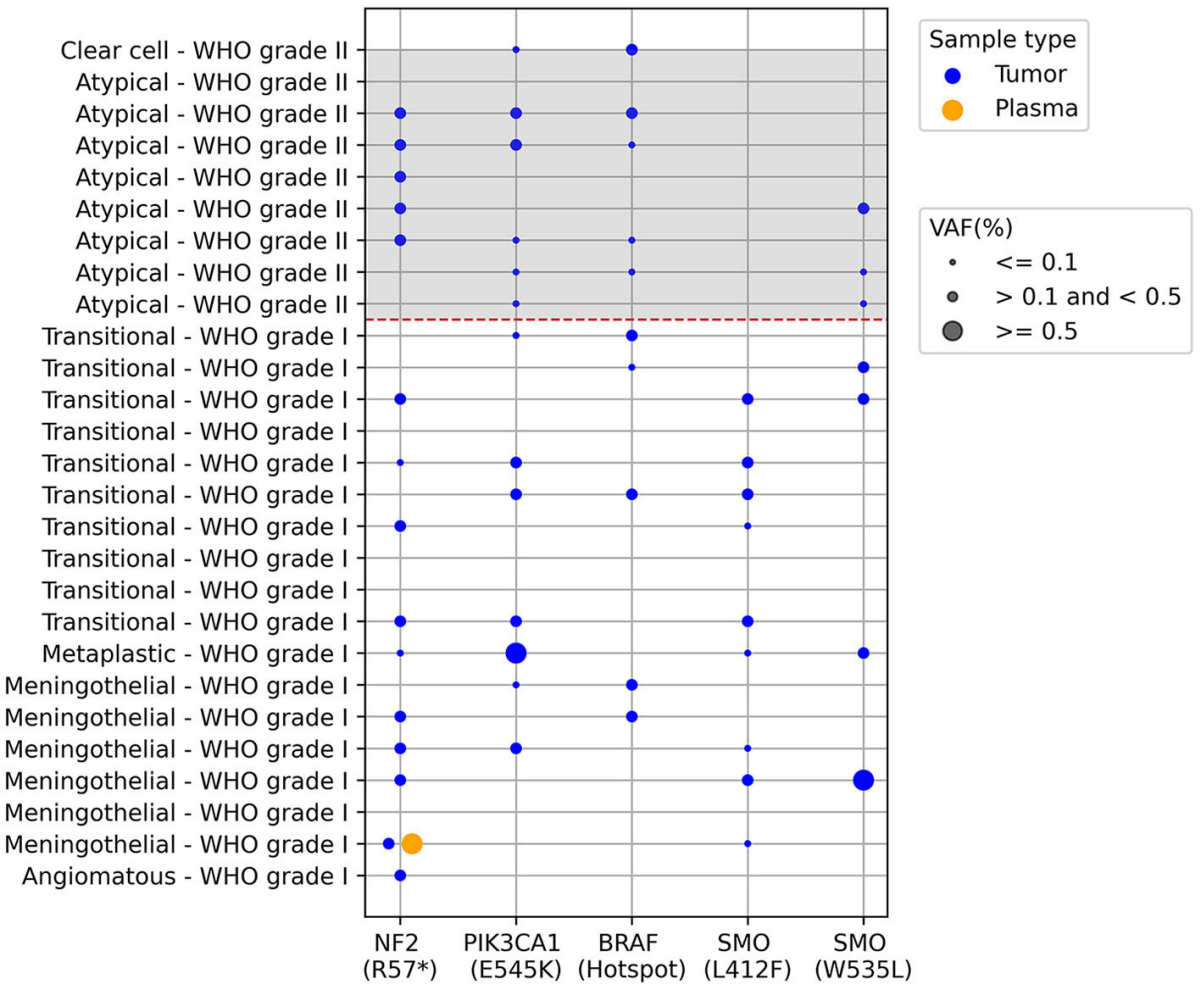
ctDNA screening in paired tumor and plasma meningioma samples using ddPCR. Each row is an individual patient and columns are different results regarding presence of mutations described below de graph, the dashed red line separates the WHO grade I from grade II meningiomas. The dots represent the presence of the mutations and dot size represents the mutant allele frequency percentage (VAF): ≤0.1; >0.1 and <0.5; ≥0.5. *BRAF* V600 hotspot mutations correspond to the following alterations: V600E, V600K, and V600R, plus wild-type. The analysis was performed in 27 samples, since one sample presented very low cfDNA for the analysis.

Plasma samples collected prior to surgery were subjected to analysis to detect mutant ctDNA using Bio-Rad's mutation-specific assays via ddPCR as described in the methods section. These assays can detect both the wild-type and mutant versions of the gene of interest. The results depicted in Figure 1 indicate distinct mutation frequencies among tumor samples removed during surgery and plasma samples obtained before surgery. The mutation incidence in tumor samples was as follows: NF2 R57^*^ (15/27), *PIK3CA E545K* (13/27), *BRAF* Hotspot mutations (10/27), *SMO L412F* (9/27), *SMO W535L* (7/27), with varying mutant allele frequencies represented by the dot sizes. In the paired plasma samples, our analysis revealed just a single instance of paired mutations, specifically impacting the *NF2* gene (NF2 R57^*^), as illustrated in Figure 1.

### Evaluation of miR-21 in the plasma

**Figure 2:**
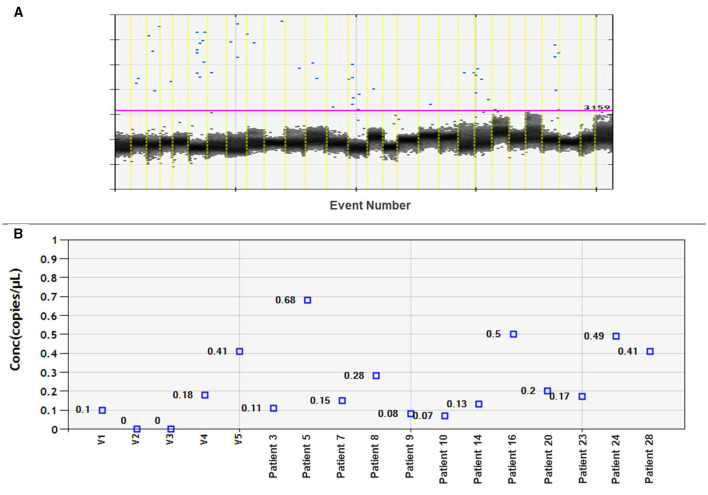
Assessment of miRNA-21 CNV analysis on meningioma plasma samples. **(A)** Fluorescence amplitude graph displays numbers of copies per well. The black cluster on the graph represents negative droplets, while the blue dots represent positive droplets. **(B)** Concentration graph of copy numbers for control samples (volunteer: V1–V5) and 12 plasma samples from meningioma patients. The remaining samples not shown on the graph did not amplify miRNA-21.

Twenty-eight plasma samples from patients with meningioma and six plasma samples from healthy volunteers were tested for miR-21 expression using ddPCR. It was possible to detect miR-21 only in 12 out of the 28 patient samples. However, there was a low detection rate, and no significant differences compared to samples from healthy volunteers (Figure 2).

### Identification of cytokines in the plasma

**Figure 3:**
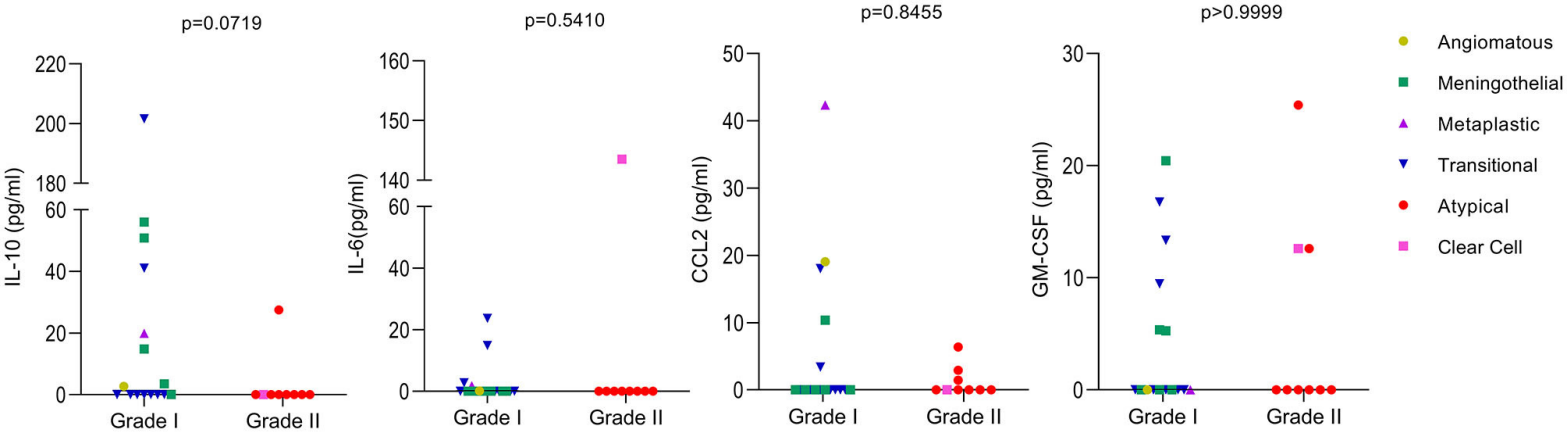
Levels of cytokines in meningioma patients. The levels of the cytokines IL-10, IL-6, CCL2, and GM-CSF were analyzed by ELISA using the plasma of 27 patients. Meningiomas were divided in two groups based on their WHO classifications grade I and II. The meningioma subtypes are identified in the figure. Values below the limit of detection were considered as zero. Mann-Whitney was used to calculate *p*-values between groups with GraphPad Prism 9.

The levels of the cytokines IL-6, IL-10, and GM-CSF, as well as the levels of the chemokine CCL2, were analyzed in the plasma of 27 patients with meningiomas (since one case had an undetermined subtype). Meningiomas were divided into grades I and II, and no significant difference was observed between them, except for IL-10, which was close to significance (*p* = 0.0719), being higher in grade I when compared to grade II. When considering different meningioma subtypes, it is evident from the results that the patient with clear cell meningioma have higher levels of IL-6 compared to the other types. Additionally, the patient with the metaplastic subtype had higher levels of CCL2 than the other patients. Unfortunately, we only had one case of each of those meningioma subtypes, necessitating further studies to investigate the relationship between these subtypes and cytokine levels (Figure 3).

## Discussion

Analyzing different types of alterations in meningioma tumors can provide valuable insights into various aspects of the tumor, including its subtype, aggressiveness, and potential treatment options. For example, the identification of specific mutations within the plasma of patients can serve as a diagnostic tool, thereby underscoring the potential of liquid biopsies as a less invasive alternative to conventional tissue biopsies for tumor profiling. Furthermore, specific mutations may be amenable to targeted therapeutic approaches, including precision medications and immunotherapies. Additionally, continuously monitoring the presence and changes in the plasma can yield significant benefits for evaluating treatment effectiveness or the potential resurgence of the disease in a non-invasive manner. Discovery of crucial immune cells and genes for the diagnosis and treatment of meningioma was recently discussed (21).

The present study analyzed plasma and FFPE tumor samples from 28 meningioma patients. Regarding the clinical variables, there was a higher prevalence of females compared to males, consistent with findings from previous studies (22, 23). Among the cases we examined, Grade I meningiomas were the most frequently represented 67.8% (19/28), while Grade II meningiomas corresponded to 32.2% of the total (9/28). It is worth noting that Grade III meningiomas were not included, which is consistent with their recognized lower incidence within meningiomas when compared to Grades I and II (23).

With advancements in both sensitivity and precision in plasma cell-free DNA (cfDNA) next-generation sequencing (NGS), it is now possible to detect somatic genomic tumor mutations with as little as 0.02% variation allelic frequency (VAF). This raises the question of whether low-frequency targetable driver mutations, defined in this context as those found below 0.2% VAF, still hold significance when additional genomic changes at higher VAF are present (24). In the present study we evaluated the DNA extracted from paired plasma and FFPE tumor samples and we found low-frequency targetable mutations from the tumors and only one analysis showed consistency between tumor and plasma samples. The observed inconsistency between plasma and tumor samples could potentially be attributed to the low concentration of cfDNA obtained from plasma (< 5 ng/reaction), a challenge commonly seen in patients with CNS tumors (25). In fact, the ctDNA concentration in plasma is notably low, constituting < 0.01% of the overall cfDNA concentration, particularly in cases of early-stage cancers (26). Moreover, the patient who exhibited the NF2 R57^*^ mutations in both plasma and tumor tissue displayed a notably higher VAF in the plasma in comparison to the tumor, an unexpected finding. While one possible explanation could be the presence of congenital Neurofibromatosis type 2, the patient's diagnostic tests did not confirm this condition. This outcome may be attributed to undisclosed factors or undiagnosed medical conditions.

In the present study we were able to identify mutations that have been described in meningioma, at a low frequency, in addition to the interesting fact that some patients presented co-occurring mutations. Unfortunately, we lack patient follow-up data to investigate the potential influence of co-occurring mutations on their prognosis and recurrence. The relevance of low VAF in the genes investigated in the present work has not been described before in meningiomas. Previously, it has been demonstrated for lung cancer patients with *EGFR T790M* treated with EGFR-TKI that there was no difference in progression-free survival when comparing low VAF (< 5%) with high VAF cases. Additionally, the same group presented a case of Non-Small Cell Lung Cancer that had *EGFR T790M* at a VAF of 3.5% that enrolled AZD9291 trial and achieved partial remission (27). So, knowledge of these low VAF in meningiomas can still prove to be useful when longer follow-up data from patients is taken into consideration.

*BRAF* mutations in meningiomas are relatively rare compared to other types of tumors, however, they can still occur (5). *BRAF V600E* mutation has been observed in one third of rhabdoid meningiomas (6), which have poor prognosis, and could be useful in the identification of these aggressive tumor subtypes as well as for the stratification of patients eligible for tentative treatment with BRAF inhibitors such as vemurafenib or dabrafenib (5). In the present analysis we observed 10/27 patients presenting mutations in this gene. The mutation was present in 27.7% grade I and 55.5% grade II meningiomas and these frequencies might be explained by the high sensitivity of the ddPCR technique as well as by the fact that other studies detect only *BRAF* V600E mutation instead of the several V600 hotspot codon mutations probed by our assay.

Mutations in the genes *NF2, SMO* and *PIK3CA* have also been reported (28, 29) using other methods of analysis, which could explain differences between our mutation frequencies and other studies frequencies. For example, although *PIK3CA* mutations have been previously described in meningioma, it has been reported that *PIK3CA* mutations frequently co-occur with *TRAF7* mutations but are mutually exclusive with *AKT1* and *SMO* mutations (29). In our analysis, seven *PIK3CA*-mutated cases, also displayed *SMO* mutations. More studies should be performed to further confirm this finding in different cohorts around the world.

Although initially suggested to be indicative of tumor progression (30), *PIK3CA* mutations are also found in low grade meningiomas (29). Our data agrees with this finding, with *PIK3CA* mutations present in both grades I and II tumors. Patients with alterations in this gene are candidates for personalized treatment with PI3K inhibitors, highlighting the potential of ddPCR for meningioma's management.

The *SMO* gene is yet another altered gene in grade I and II meningiomas whose mutations activate the Hedgehog signaling pathway (31). Regarding prognosis, grade I olfactory groove meningioma with *SMO* mutations seem to have an unfavorable outcome (32), but none of our patients' displayed tumors in this anatomical location. Additionally, since inhibitors of this pathway hold great promise for the treatment of meningiomas harboring *SMO* mutations, ddPCR mutational analysis in tumor tissue has, once more, implications for targeted-individualized therapy in the *SMO*-mutated context.

Regarding the distribution of mutations according to tumor grades and subtypes, all mutations were present in most subtypes and grades analyzed, except for *SMO L412F* which was absent in grade II patients, which could be partly due to the reduced size of our cohort. In addition, a study have suggested that *SMO* mutations are more frequent in grade I meningiomas than in grade II (32).

Overall, the low mutation frequencies observed in the present study could be explained by the high sensitivity of the ddPCR technique. Using other techniques such as Sanger sequencing and NGS, detecting mutations at levels below 1% can be challenging (33, 34), potentially leading to the omission of mutations in patients who might be incorrectly classified as having wild-type genes. Indeed, it seems reasonable to assume that at least part of the meningioma cases previously believed to be caused by yet unrecognized genetic factors (35) might in fact contain *BRAF/NF2/PIK3CA/SMO* mutations at frequencies inferior to the limits of traditional detection methods. Therefore, ddPCR has the potential to enhance mutation detection in this context, opening new opportunities for studying the spectrum of low-frequency mutations and enhancing tumors characterization. This could usher in a new era of research focused on these previously elusive genetic variations.

Regarding miRNAs, miR-21 was the initial miRNA identified in relation to cancer, and its positive expression has been documented across a range of cancer types, strongly associated with its oncogenic characteristics (13). It exerts influence over various cellular processes, spanning from tumor initiation to cell death. Furthermore, its association with resistance to cancer treatment has been well-established (9). In the context of meningiomas, miR-21 has been detected particularly in more advanced tumor cases (14, 15). In our research, while we were able to detect miR-21 expression in the plasma of meningioma patients, there was no notable discrepancy when compared to samples obtained from healthy volunteers. This lack of distinction may be accounted for by the predominantly benign nature of meningiomas (20). The reasons behind the extensive variation in miRNA gene expression between malignant cells and normal cells can be attributed to the positioning of these genes in genomic regions linked to cancer, epigenetic mechanisms, and changes in the miRNA processing apparatus (36).

Regarding the cytokines identified in plasma, although we have not observed a significant result, the data was still interesting when meningiomas were divided into WHO grades I and II. We observed that IL-10 was close to significance (*p* = 0.0719), being higher in grade I when compared to grade II and that IL-6 was only expressed in the Clear cell subtype (grade II) and in the transitional subtype (grade I), with higher levels in the Clear cell subtype than in the transitional subtype. IL-6 was shown to be directly involved in tumor immunosuppression influencing the generation of myeloid-derived suppressor cells and tumorigenesis *per se* (37), being expected to be more pronounced with tumor progression. In addition, high serum levels of IL-6 were shown to predict poor response to therapy in advanced renal cell carcinoma (38). As mentioned previously, the incidence of grade II is lower than that of grade I, so there were more samples tested for grade I. Unfortunately, when dividing into subtypes we had insufficient samples for each analysis, being intricate to take any further conclusions.

The present work has certain limitations stemming from the lack of comprehensive clinical data and follow-up information for the patients, making it challenging to correlate the obtained results with each patient's prognosis. Additionally, improving the yield of cfDNA from the extractions would have been beneficial. We believe that by utilizing higher cfDNA concentrations in the ddPCR analysis, we could have enhanced the likelihood of identifying more matched plasma-tumor mutations. Unfortunately, we were unable to establish a consistent conclusion regarding the impact of miR-21 in the present analysis. We also hold the view that a study involving a larger patient cohort will be vital to more conclusively validate the significance of circulating cytokines in the context of meningioma.

## Conclusions

In conclusion, liquid biopsy emerges as a promising non-invasive diagnostic and monitoring method, holding potential for the diagnosis of central nervous system tumors like meningiomas. While our study did not demonstrate a significant concordance in plasma-tumor mutations, it was intriguing to note that the ddPCR technique can be instrumental in detecting mutations with a low allelic frequency within solid tumors. Additionally, the assessment of plasma cytokines suggests potential utility in meningioma patients, especially taking in consideration the distinct meningioma subtypes. In addition, it would be useful to study a larger cohort of miRNAs since miR-21 was identified in some patients. To fully grasp its clinical significance in meningiomas, further extensive research, including large cohort analyses, is imperative.

## Data availability statement

The raw data supporting the conclusions of this article will be made available by the authors, without undue reservation.

## Ethics statement

The studies involving humans were approved by Ethics Committee of Instituto Estadual do Cérebro Paulo Niemeyer. The studies were conducted in accordance with the local legislation and institutional requirements. The participants provided their written informed consent to participate in this study.

## Author contributions

VA: Conceptualization, Data curation, Formal analysis, Investigation, Methodology, Writing – original draft, Writing – review & editing. RL: Data curation, Formal analysis, Investigation, Methodology, Writing – review & editing, Writing – original draft. MH: Investigation, Methodology, Writing – original draft, Writing – review & editing. AC: Investigation, Methodology, Writing – original draft, Writing – review & editing. FA: Data curation, Formal analysis, Methodology, Writing – review & editing, Visualization. LC: Writing – review & editing, Visualization. SD: Writing – review & editing. PN: Visualization, Writing – review & editing. VM-N: Conceptualization, Funding acquisition, Resources, Supervision, Writing – review & editing, Visualization, Investigation.
